# Statistical Optimization of Culture Conditions for Protein Production by a Newly Isolated *Morchella fluvialis*

**DOI:** 10.1155/2019/7326590

**Published:** 2019-12-23

**Authors:** Zahra Rahgo, Hamid reza Samadlouie, Shideh Mojerlou, Kambiz Jahanbin

**Affiliations:** ^1^Shahrood University of Technology, Faculty of Agriculture, Department of Food Science and Technology, Shahrood, Iran; ^2^Department of Horticulture and Plant Protection, Faculty of Agriculture, Shahrood University of Technology, P. O. Box: 3619995161, Shahrood, Iran

## Abstract

*Morchella* fungi are considered a good source of protein. The ITS region was used to identify *Morchella* isolated in the northern region of Iran. The isolated fungus was very similar to *Morchella fluvialis*. *M. fluvialis* was first isolated in Iran. Dried biomass of *M. fluvialis* contained 9% lipids and 50% polysaccharides. Fatty acid profiles of lipids of *M. fluvialis* are mainly made up of linoleic acid (C18:2) (62%), followed by palmitic acid (C16:0) (12%). Testosterone (TS) was also detected (0.732 ng/dry weight biomass (DWB)) in the hormone profile of this new isolated species. Then, various protein and carbon sources as variable factors were applied to identify the key substrates, which stimulated protein production using the one-factor-at-a-time method. Key substrates (glucose and soybean) were statistically analyzed to determine the optimum content of the protein and DWB accumulation using response surface methods. The highest protein content (38% DWB) was obtained in the medium containing 80 g/l glucose and 40 g/l soybean powder. Total nutritionally indispensable amino acids and conditionally indispensable amino acids constitute 55.7% crude protein. That is to say, these adequate quantities of essential amino acids in the protein of *M. fluvialis* make it a good and promising source of essential amino acids for human diet.

## 1. Introduction

*Morchella* are edible fungi belonging to the class Ascomycetes and are closely related to simpler cup fungi in the order Pezizales. Ridges with pit frameworks of fungi cap creating a honeycomb structure were frequently considered to recognize these distinguished fungi. These precious and delicious fungi were discovered in China, India, Turkey, the Himalayas, and Pakistan. For years, the number of species was the subject of taxonomic controversy, while current phylogenetic trees indicated that seventy species of *Morchella* have been recognized all over the world. Various research studies have been conducted on phylogeny, biogeography, taxonomy, and nomenclature of this genus to identify new species all over the world. In spite of the fact that the primary trait of *Morchella* species is the high continental endemism and provincialism [1, 2], transcontinental species were also discovered [3–6]. In addition, the inconsistent ecological potential of *Morchella* species produced symbiotic, endophytic, and saprotrophic abilities [7–11]. Morchellaceae family has featured a wide diversity of bioactive components with curative properties [12]. High protein content along with unique flavor and medicinal properties [13] is the main observable characteristic to consider this species as a famous edible mushroom [14]. These fungi were found to have antiviral, antioxidative, and anticancer properties [15–17]. Submerge fermentation is advantageous due to cost-effectiveness, low temperature requirement, effective contamination control, and shorter fermentation time. With these promising factors, submerge fermentation is frequently used to enhance the vital components of *Morchella* [14, 18, 19]. That is to say, various research studies on *Morchella* were conducted in submerge fermentation (SMF) to produce vital components such as antioxidants and polysaccharides [14, 20, 21]; SMF has rarely been applied to optimize protein production. After the fungi were isolated, they were identified with the PCR method, and fatty acid profiles, hormone profile, total protein content, and polysaccharide content of the isolated fungi were analyzed. SMF was used to produce proteins; the protein content was optimized using the one-factor-at-a-time method and RSM. Finally, the amino acid profile of *Morchella* was analyzed at the optimal condition, which had the highest protein content.

## 2. Materials and Methods

### 2.1. Fungi Culture

The mushroom was found at the lower elevations of mountainous areas in the northern region of Iran, Gorgan, Golestan Province. The fungus fruit was split using a sterilized surgical blade, and then a patch of the fruit body which had the least connection with its surrounding was removed and put in an enriched PDA medium containing mineral elements.

### 2.2. Molecular Identification

To identify the fungal species, the sample was first incubated in a seed culture medium. Then, DNA was extracted using a DNA extraction kit (K721, Thermo, USA). Quantity and quality of the extracted DNA were determined by using a spectrophotometer and agarose gel, respectively. The ITS region (ITS1, 5.8S, ITS2) was amplified with the universal primers ITS1F (5′-GCATATCAATAAGCGGAGGAAAAG-3′) [22] and ITS4 (5′-TCCTCCGCTTATTGATATGC-3′) [23], with an initial denaturation for 5 min at 94°C and then 30 cycles consisting of denaturation at 94°C for 30 sec, annealing at 55°C for 30 sec, and extension at 72°C for 1 min, and a final extension at 72°C for 5 min was applied. Sterilized distilled water was used as a negative control. PCR products were electrophoresed in a 1.2% (w/v) agarose gel. In order to assure the accurate identification, the elongation factor EF1*-α* gene using 1577F (5′-CARGAYGTBTACAAGATYGGTGGG-3′), 1567RintB (5′-ACHGTRCCRATACCACCRAT-3′), and 2212R (5′-CCRAACRGCRACRGTYYGTCTCAT-3′) primers [1, 24] was also served. PCR conditions were identical to previously mentioned method, with the exception of slight fall in annealing temperature at 62°C. Sequencing was done by Takapouzist Company (http://www.takapouzist.com). Blast searching of ITS sequences was done, and the sequences were aligned using Mega 6.0 software. The clustering method UPGMA was used to draw the phylogeny with the aid of the Mega 6.0. 1000 bootstrap replicates. The consensus tree was also drawn by Mega 6.0.

### 2.3. Seed and Fermentation Media

Isolated fungus was incubated in an enriched PDA medium containing 10 grams per liter glucose and 2 grams per liter soybean powder at 25°C for 10 days. Seed media were used in the fermentation media containing various carbon and protein sources along with KH_2_PO_4_ (3 g/L), MgSO_4_·7H_2_O (0.5 g/L), ZnSO_4_·7H_2_O (0.3 g/L), FeSO_4_·7H_2_O (0.2 g/L), and KNO_3_ (0.25 g/L) as mineral elements. The temperature of 20°C for five days at 180 rpm and pH 6 were constant factors.

### 2.4. Analytical Methods

To extract polysaccharides, mashed dry mycelia were immerged into hot water (1 : 20, W/V ratio) at 60°C for 3 h. The polysaccharides were analyzed by the method of phenol-sulfuric acid [25]. Fatty acids were analyzed by gas chromatography (Unicam 4600, England) with a flame ionization detector (FID) [26]. Amino acids were analyzed and determined by ion-exchange chromatography with postcolumn derivatization with ninhydrin. Amino acids were oxidized with performic acid, which was neutralized with Na metabisulfite. Amino acids were liberated from the protein by hydrolysis with 6 N HCl for 24 hr at 110°C and quantified with the internal standard by measuring the absorption of reaction products with ninhydrin at 570 nm [27, 28]. Freeze-dried biomass of the fresh fungi tissue was used to extract hormones, and a completely robotized immunochemical analyzer Cobas e 411 was used to recognize the quantity and quantity of fungi hormone profiles.

## 3. Results

**Figure 1:**
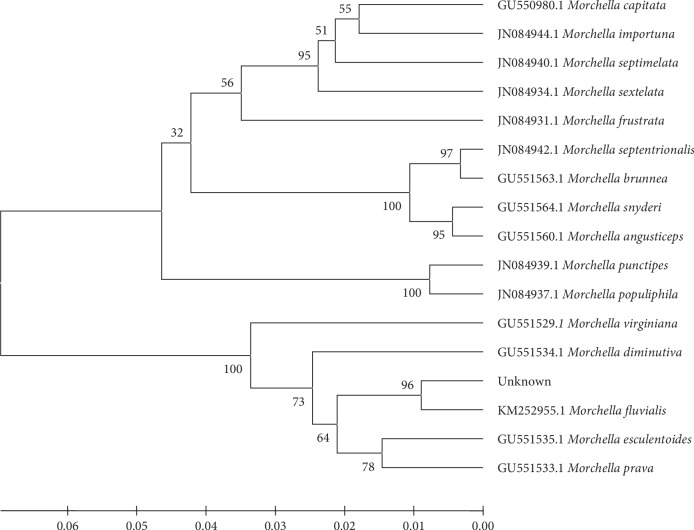
Phylogenetic consensus tree based on EF1-*α* gene sequences calculated by the UPGMA method. Isolates of *Morchella* obtained in this study are “unknown.” Numbers on nodes indicate bootstraps (*n* = 1000).

The low growth rate and particularly a thick mycelium structure were the main characteristics of *Morchella* that were used to differentiate them from other fungal contaminations. EF1-*α* and ITS region genes were used for microbial species identification, and the results indicated that the isolated fungus had the highest similarity (99%) to *M. fluvialis* (Figure 1). Sequences of the ITS region of the isolated fungi were registered in the NCBI database with the accession number MK011022; this new isolate of *M. fluvialis* has been first introduced from Iran.

### 3.1. Fatty Acid Profiles and Polysaccharide Content of *M. fluvialis* Fruit

**Figure 2:**
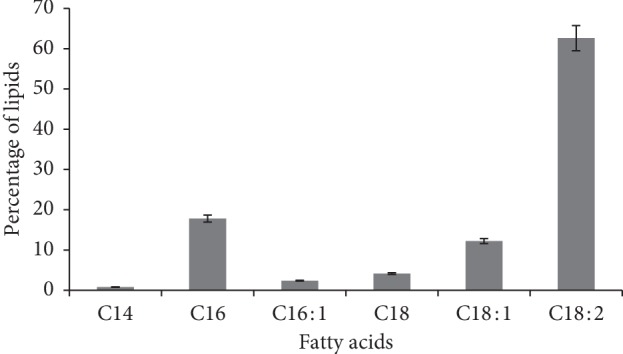
Fatty acid profiles of *M. fluvialis*' lipid.

Linoleic acid (C18:2) was predominant (62%) in *M. fluvialis* lipid, followed by palmitic acid (C16:0) (12%) (Figure 2). Polysaccharide content of this fungus was 50% DWB, which is 20% lower than that of the *M. esculenta* species [29].

### 3.2. Hormone Analysis of *M. fluvialis* Tissue

The chemical productions of testosterone (TS) was chemically synthesized from androst‐4‐ene‐3,17‐dione (AD) [30]. Some varieties of microorganisms including yeasts [31–34] and filamentous fungi [35] were able to enzymatically convert AD to TS. Among various microbial resources of TS production, fungal species are able to produce a wide variety of enzymes which engender high quantity of sterane skeleton [35].  Table 1 indicates that the fruit of this fungus was potentially a good source of various hormones. TS was observed in *M. fluvialis* hormone profile, so this species could be used as a good and reliable source of this vital component.

### 3.3. Investigating the Effects of Various Substrates on DWB and Protein Content of *M. fluvialis* Using the “One-Factor-At-A-Time” Method

#### 3.3.1. Nitrogen Sources

**Figure 3:**
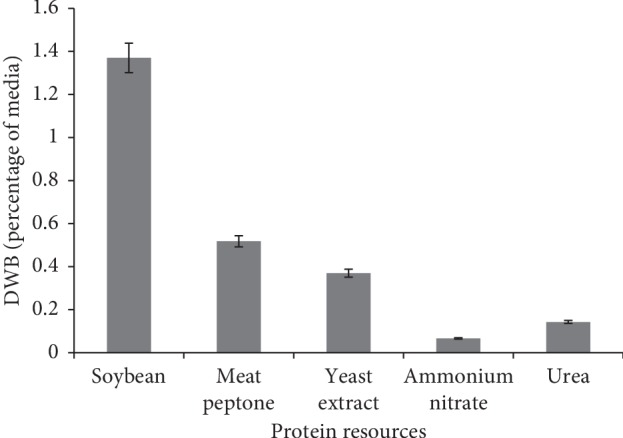
Effect of various protein sources on DWB.

**Figure 4:**
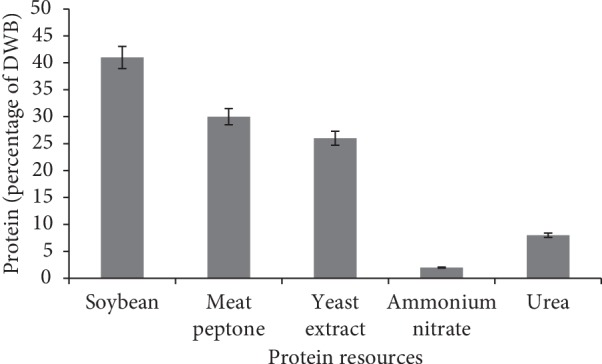
Effect of various protein sources on *M. fluvialis*' protein.

Various protein sources were used as variable factors. The highest DWB content was obtained in the medium containing soybean protein, while the lowest amount of DWB was obtained in the medium that had the inorganic nitrogen sources like ammonium nitrate and urea (Figure 3). Various researchers revealed the fact that the protein source had a significant effect on DWB accumulation in fungal species [36, 37]. Soybean protein had greater impact on DWB than the yeast extract media (Figure 4). The results were in agreement with those of Park et al. [36], who reported that soy protein was ranked as an appropriate medium for secondary metabolic productions. Park et al. [36] reported that gradual consumption of the low-soluble soybean powder protein was the main factor, which stimulated secondary metabolic productions.

#### 3.3.2. Carbon Sources

**Figure 5:**
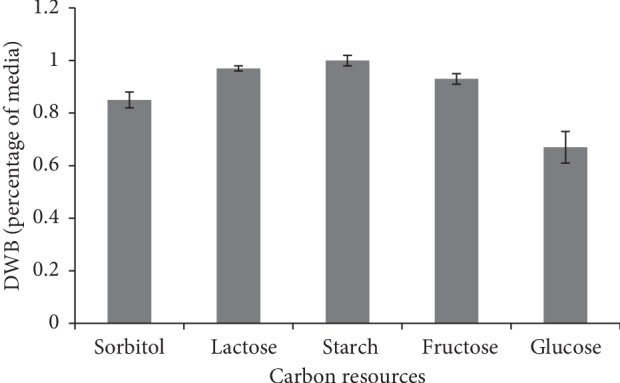
Effect of various carbon sources on DWB.

**Figure 6:**
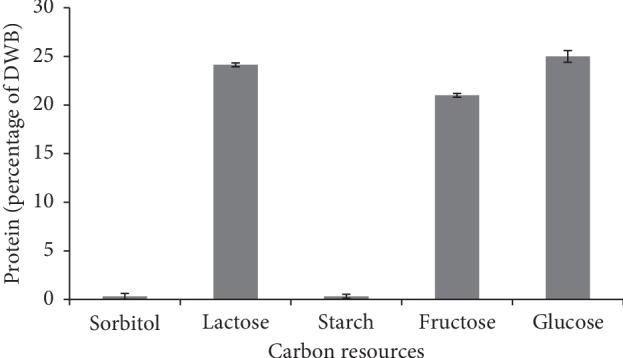
Effect of various carbon sources on *M. fluvialis*' protein.

Carbon sources as variable factors were examined, and in each medium, 20 g/l soybean as the best protein resource inducing more protein production was added. The starch substrate supported the highest DWB accumulation. Zhang et al. [38] reported that *Morchella esculenta* was the good source of enzymes, which in optimal conditions could assimilate starch by reducing it from 64.5% to 23.5%. The least amount of DWB was observed in the medium containing glucose (Figure 5). However, the high amount of protein accumulation was observed in this medium. It could be concluded that glucose substrate stimulated *M. fluvialis* to produce high content of protein instead of DWB accumulation (Figures 5 and 6). The results of the one-factor-at-a-time method revealed that the soybean protein and glucose substrate were of vital importance to induce *M. fluvialis* for the highest protein production.

### 3.4. Optimization by RSM

Optimization is regarded as a scientific trend to attain a mathematical model for predicting the correlation between responses and independent variable factors. The statistical method RSM has less experimentation than a complete factorial design [39]. The face-centered central composite design (FCCCD) of the RSM served to determine the appropriate quantity of the each aforementioned factor and analyze their interactions on protein and DWB accumulation. A wide range of these two key factors (glucose and soybean powder) was applied according to the previous studies to investigate the effect of each of the factors and their interactions on the quantity of the responses.

The central composite design of the response surface method is provided in Table 2. The results indicated that the soybean protein had a great impact on the DWB and protein accumulation than glucose. Thus, with a slight increase in the protein content, biomass sharply increased (runs 6 and 9). Any increase in glucose content at a given constant level of soybean also produced a rise in DWB and protein content of DWB (runs 2 and 9). The results obtained by FCCCD were then surveyed by the analysis of variance (ANOVA), and the results were applied to fit a second-order polynomial equation. As shown in (Tables 3 and 4) the linear effects of soybean powder and glucose on the amount of the DWB and protein were significant (*P* < 0.01). The interactions of glucose and soybean powder were not significant for both responses (*P* > 0.01). The quadrant effect of the carbon source on the quantity of protein production was significant (*P* < 0.05) (Table 4). The numerical value of the coefficient of determination (*R*^2^) for both protein and DWB was 0.98, indicating the degree of matching the data in the regression model. It could be concluded that the regression models were able to well calculate and predict the correlations between culture conditions (glucose and soybean powder) and responses (protein and DWB content). Also, the lack of fit of the final model was nonsignificant, which indicated a good fit of the model.

The fitted equation of DWB (Y1) and protein production content (Y2) over the level of glucose and soybean powder was indicated as follows:

(1) $$\begin{array}{c} \text{DWB}=-3.54109+0.063174\times \text{glucose}+0.159877\times \text{soybean}+9E-05\times \text{glucose}\times \text{soybean}-0.00048\times {\text{glucose}}^{2}-0.00185\times {\text{soybean}}^{2},\\ \text{protein}=-47.86483+1.29728\times \text{glucose}+1.66738\times \text{soybean}-5.83750E-003\times \text{glucose}*\text{soybean}-7.82031E-003\times {\text{glucose}}^{2}-0.011281\times {\text{soybean}}^{2}. \end{array}$$

**Figure 7:**
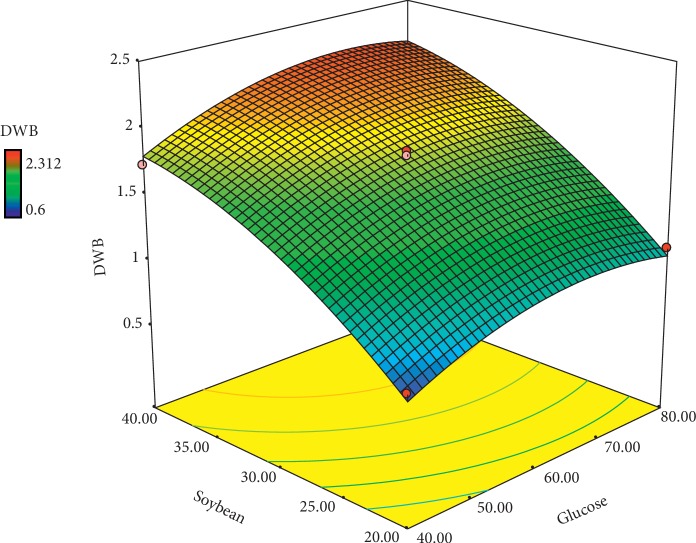
Response surface curve for DWB production by *M. fluvialis*.

**Figure 8:**
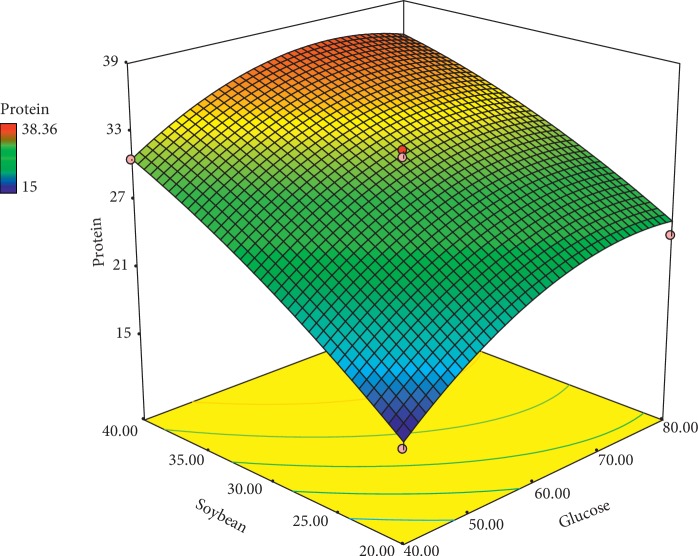
Response surface curve for protein production by *M. fluvialis*.

Figure 7 shows the effect of glucose and soybean powder on the quantities of DWB. The results showed that an increase in glucose and soybean powder stimulated DWB production. The highest amount of DWB was obtained at the high quantity of glucose (60–70 g/l) and soybean powder (40 g/l). Jin et al. [37] reported that the protein substrate had a positive effect on DWB production [37].  Figure 8 shows the effect of different levels of glucose and soybean powder on protein accumulation. Increase in soybean powder (40 g/l) and glucose content (68 g/l) produced a high accumulation of protein in DWB. Research conducted by Reihani and Khosravi-Darani [40] showed that the nitrogen source had a significant effect on protein production in single-cell protein fungi. The optimal predicted values for variable factors were 68 g/l glucose and 40 g/l soybean powder to produce the highest amount of the protein (36.9%) content in DWB.

#### 3.4.1. Verification of Optimal Conditions

In order to verify the model, the optimum values of the DWB and protein productions predicted by RSM were experimentally verified. RSM predicted that the appropriate culture condition of the protein production was 68 grams per liter of glucose and 40 grams per liter of soybean powder, and an appropriate culture condition for DWB production was 69.58 and 40 g/l glucose and soybean powder, respectively. After 5 days of fermentation, the actual protein content in the mentioned media was 38% and the DWB content was 2.2%. With comparison of these two predicted values, the error rate was 2% and 1% for protein and DWB production, respectively. Anupama and Ravindra [41] indicated that the best proportion for the maximum protein production was 1.38 parts carbon to 1 part nitrogen; the ratio was 1.75/1 in the present study.

### 3.5. Amino Acid Analysis

*M. fluvialis*' crude protein (CP) and amino acid profile of proteins at the optimal conditions after five days of fermentation was analyzed. The CP content standardized to 88% was 37.90. Amino acid analysis standardized to 88% dry weight matter indicated that the predominant amino acid was glutamic acid (12.434%) and aspartic acid (9.29%). Importantly, nutritionally indispensable amino acids like phenylalanine (4.769), leucine (6.297%), valine (4.811), threonine (4.05%), lysine (3.096%), methionine + cystine (2.634%), histidine (1.833), and methionine (1.212%) comprised 28.7% of the total protein, and conditionally indispensable amino acids like glycine (4.276%), proline (4.699%), arginine (3.999%), and cystine (1.422%) comprised 14.4% of the total protein (Table 3).

## 4. Discussion

The phylogenetic tree revealed that the isolated fungus was *M. fluvialis* belonging to Morchellaceae family, which was first reported and disassembled by Clowez et al. [42]. This fungus is similar to *M. esculenta* [42] which was first isolated in Spain. In spite of the similarity between *M. fluvialis* and *M. esculenta*, research studies have rarely been done on protein production using this fungus. Various research studies indicated *M. esculenta* had a high potential for protein production [43]. In line with the present research, LeDuy et al. [44] showed that the amount of protein in this fungus reached 32.7% of the DWB. The digestibility of the edible fungi protein ranged between 72% and 84%, which has been the main feature of this edible fungi species [45]. Substrate components, fermentation conditions, and the fungal species were among vital factors impacting the amino acid profile of protein and quantity of protein [46]. Roy and Samajpati [47] reported that the amount of protein in *M. esculenta* and *M. deliciosa* was 34.7% and 29.16%, respectively. The crude protein of the fungus was lower than that of the meat, while it was higher than that of most of the food, including milk [48]. The protein content of *M. esculenta* was typically between 19% and 35% compared with rice (7.3%), wheat (12.7%), corn (9.4%), and soybeans (38.1%) [49, 50]. Protein accumulation reached 39% of DWB at optimal conditions, which constituted a significant proportion of *M. fluvialis*. In edible fungi, lipid content is generally lower than carbohydrate and protein content [51]. Research has shown that lipids obtained from edible fungi have more structural unsaturated fatty acids [29], with linoleic acid, a structural fatty acid [52], being predominant. Yilmaz et al. [53] reported that unsaturated fatty acids are predominant in edible fungi. Heleno et al. [29] reported that the unsaturated fatty acids were higher in *M. esculenta* species than saturated fatty acids. Linoleic, oleic, and palmitic were the predominant fatty acids in the lipid content of *M. esculenta*. In comparison with research studies done, fatty acids of *M. fluvialis* had high similarity to *M. esculenta*. Furthermore, linoleic acid was the predominant fatty acid in both fungi.

Hasan [54] and Fernández Cabezón et al. [55] reported that various fungi species like *Aspergillus flavus*, *A. ochraceus*, *Gibberella zeae*, *Cladosporium cladosporioides*, *Penicillium funiculosum*, and *P. rubrum* were capable of producing high amount of hormones. Many research studies indicated that gut microflora had a great role in estrogen and phytoestrogen production [56–58]. This research indicated that the fresh tissue of *M. fluvialis* was a good source of TS. Verma et al. [59] reported that edible fungi had the high quantity of essential amino acids with higher similarity to meat protein. In 1976, Hayes and Haddad [51] reported that the essential amino acids explored in fungi species were of vital importance to use as dietary supplements. At optimal conditions, the DWB of *M. fluvialis* was made up of 38% protein. Considering the 77.38% of the total protein including NH_3_, the total nutritionally indispensable amino acids and conditionally indispensable amino acids comprised 43.1% crude protein, which make *M. fluvialis* as a good source of essential amino acids.

## 5. Conclusion

The appropriate amount of hormones, polysaccharides, and valuable proteins was the main feature of this new isolated fungus. Phylogenetic tree revealed that this species was *M. fluvialis* which was first isolated in Iran. The protein content of *M. fluvialis* was 36% DWB. The nutritionally indispensable amino acids and conditionally indispensable amino acids made up 28.7% and 14.4% of the total protein, respectively, making this fungus a vital source of essential amino acids.

## Figures and Tables

**Table 1:** Hormone profile of *M. fluvialis* tissue.

Test	Result
Dehydroepiandrosterone sulfate (DHEAS)	6 nG/DWB
Estrogen (E2 111)	62.88 pg/DWB
Follicle-stimulating hormone (FSH)	96 mIu/DWB
Luteinizing hormone (LH)	4.40 mIu/DWB
Prolactin (PRL)	0.312 ng/DWB
Progesterone (PROG)	0.68 ng/DWB
Testosterone (TS)	0.732 ng/DWB

**Table 2:** Results of FCCCD using two variables showing observed responses.

	Glucose (g/L)	Soybean (g/L)	Protein	DWB
1	31.71	30	20	1.21
2	40	20	15	0.8
3	60	30	31.5	1.836
4	60	15.85	21	0.6
5	88.28	30	31.36	1.673
6	80	40	35	2.104
7	60	30	30.86	1.8
8	40	40	30.67	1.732
9	80	20	24	1.1
10	60	44.14	38.36	2.312

**Table 3:** Results of amino acid analysis/total contents after hydrolysis of *M. fluvialis*' protein.

Parameter	Content (% as is)	Content (%)^*∗*^	Content (% in CP)
Methionine	0.381	0.363	1.212
Cystine	0.447	0.425	1.422
Methionine + cystine	0.828	0.788	2.634
Lysine	0.973	0.926	3.096
Threonine	1.273	1.211	4.05
Arginine	1.257	1.196	3.999
Isoleucine	1.304	1.241	4.149
Leucine	1.979	1.883	6.297
Valine	1.512	1.439	4.811
Histidine	0.576	0.548	1.833
Phenylalanine	1.499	1.426	4.769
Glycine	1.344	1.279	4.276
Serine	1.302	1.239	4.143
Proline	1.477	1.405	4.699
Alanine	1.502	1.429	4.779
Aspartic acid	2.92	2.778	9.29
Glutamic acid	3.908	3.718	12.434
NH_3_	0.666	0.634	2.119
Total including NH_3_	24.32	23.139	77.378

^*∗*^DMS: values standardized to a dry matter of 8%; CP = crude protein, based on Dumas combustion method (CP factor = 6.25).

**Table 4:** ANOVA parameters of the models fitted for DWB (B) and protein (P) response.

Source	Sum of squares (B)	Sum of squares (P)	df (B)	df (P)	Mean square (B)	Mean square (P)	*F* value (B)	*F* value (P)	*P* value, Prob > *F* (B)	*P* value, Prob > *F* (P)
Model	2.821581	486.3946	5	5	0.564316	97.27892	73.91291	61.99206	0.0005	0.0007
A-glucose	0.220043	108.0117	1	1	0.220043	108.0117	28.82082	68.83163	0.0058	0.0012
B-soybean	2.373077	327.9456	1	1	2.373077	327.9456	310.8205	208.9869	<0.0001	0.0001
AB	0.001296	5.452225	1	1	0.001296	5.452225	0.169747	3.47449	0.7015	0.1358
A^2^	0.168411	44.73219	1	1	0.168411	44.73219	22.05817	28.50608	0.0093	0.0059
B^2^	0.155929	5.817902	1	1	0.155929	5.817902	20.42325	3.707522	0.0107	0.1265
Residual	0.03054	6.276863	4	4	0.007635	1.569216				
Lack of fit	0.029892	6.072063	3	3	0.009964	2.024021	15.37629	9.882915	0.1848	0.2287
Pure error	0.000648	0.2048	1	1	0.000648	0.2048				
Core total	2.85212	492.6715	9	9						

## Data Availability

The original data used to support the findings of this study are included within the article.

## References

[1] O’Donnell K., Rooney A. P., Mills G. L., Kuo M., Weber N. S., Rehner S. A. (2011). Phylogeny and historical biogeography of true morels (*Morchella*) reveals an early cretaceous origin and high continental endemism and provincialism in the holarctic. *Fungal Genetics and Biology*.

[2] Olney R. (1995). *A Provencal Table*.

[3] Du X.-H., Zhao Q., O’Donnell K., Rooney A. P., Yang Z. L. (2012). Multigene molecular phylogenetics reveals true morels (*Morchella*) are especially species-rich in China. *Fungal Genetics and Biology*.

[4] Richard F., Bellanger J.-M., Clowez P. (2015). True morels (*Morchella*, Pezizales) of Europe and North America: evolutionary relationships inferred from multilocus data and a unified taxonomy. *Mycologia*.

[5] Loizides M., Bellanger J.-M., Clowez P., Richard F., Moreau P.-A. (2016). Combined phylogenetic and morphological studies of true morels (*Pezizales, Ascomycota*) in Cyprus reveal significant diversity, including *Morchellaar butiphila* and *M. disparilis* spp. nov. *Mycological Progress*.

[6] Loizides M. (2017). Morels: the story so far. *Field Mycology*.

[7] Buscot F., Roux J. (1987). Association between living roots and ascocarps of Morchella rotunda. *Transactions of the British Mycological Society*.

[8] Buscot F., Read D. J., Lewis D. H., Fitter A. H., Alexander I. J. (1992). Mycorrhizal succession and morel biology. *Mycorrhizas in Ecosystems*.

[9] Dahlstrom J. L., Smith J. E., Weber N. S. (2000). Mycorrhiza-like interaction by Morchella with species of the Pinaceae in pure culture synthesis. *Mycorrhiza*.

[10] Tedersoo L., May T. W., Smith M. E. (2010). Ectomycorrhizal lifestyle in fungi: global diversity, distribution, and evolution of phylogenetic lineages. *Mycorrhiza*.

[11] Stefani F. O. P., Sokolski S., Wurtz T. L. (2010). *Morchella tomentosa*: a unique belowground structure and a new clade of morels. *Mycologia*.

[12] Litchfteld J. H., Vely V. G., Overbeck R. C. (2006). Nutrient content of morel mushroom mycelium: amino acid composition of the protein. *Journal of Food Science*.

[13] Burnett G. T. (1835). Outlines of botany: including a general history of the vegetable kingdom. *Which Plants Are Arranged According to the System of Natural Affinities*.

[14] Meng F., Zhou B., Lin R. (2010). Extraction optimization and in vivo antioxidant activities of exopolysaccharide by *Morchella esculenta* SO-01. *Bioresource Technology*.

[15] Nitha B., Janardhanan K. K. (2008). Aqueous-ethanolic extract of morel mushroom mycelium *Morchella esculenta*, protects cisplatin and gentamicin induced nephrotoxicity in mice. *Food and Chemical Toxicology*.

[16] Nitha B., De S., Adhikari S. K., Devasagayam T. P. A., Janardhanan K. K. (2010). Evaluation of free radical scavenging activity of morel mushroom, *Morchella esculenta* mycelia: a potential source of therapeutically useful antioxidants. *Pharmaceutical Biology*.

[17] Elmastas M., Turkekul I., Ozturk L., Gulcin I., Isildak O., Aboul-Enein H. (2006). Antioxidant activity of two wild edible mushrooms (Morchella vulgaris and Morchella esculanta) from North Turkey. *Combinatorial Chemistry & High Throughput Screening*.

[18] Fang-cao C., Xing-rong L., Fang-he T., Qiang L. (2004). Studies on the biological characteristics of Morchella conica. *Southwest China Journal of Agricultural Sciences*.

[19] Li J., Zhang Y., Qiu D. (2004). Effects of different factors on spore germination and mycelia growth in *Morchella esculenta*. *Journal of Hebei University for Science and Technology*.

[20] Sun X., Wu S. L. (2000). Submerged fermentation of mycelia *Morchella esculenta*. *Jiangsu Food Fermentation*.

[21] Xu H., Sun L.-P., Shi Y.-Z., Wu Y.-H., Zhang B., Zhao D.-Q. (2008). Optimization of cultivation conditions for extracellular polysaccharide and mycelium biomass by *Morchella esculenta* As51620. *Biochemical Engineering Journal*.

[22] Gardes M., Bruns T. D. (1993). ITS primers with enhanced specificity for basidiomycetes application to the identification of mycorrhizae and rusts. *Molecular Ecology*.

[23] White T. J., Bruns T., Lee S., Taylor J. (1990). Amplification and direct sequencing of fungal ribosomal RNA genes for phylogenetics. *PCR Protocols*.

[24] Rehner S. A., Buckley E. (2005). A *Beauveria* phylogeny inferred from nuclear ITS and EF1-*α* sequences: evidence for cryptic diversification and links to *Cordyceps* teleomorphs. *Mycologia*.

[25] Chaplin M. F., Kennedy J. F. (1994). *Carbohydrate Analysis: A Practical Approach*.

[26] Metcalfe L. D., Schmitz A. A., Pelka J. R. (1966). Rapid preparation of fatty acid esters from lipids for gas chromatographic analysis. *Analytical Chemistry*.

[27] Llames C. R., Fontaine J. (1994). Determination of amino acids in feeds: collaborative study. *Journal–Association of Official Analytical Chemists*.

[28] Commission Directive (1998). Establishing community methods for the determination of amino acids, crude oils and fats, and olan-quindox in feeding stuff and amending directive 71/393/EEC, annex part A. determination of amino acids. *Official Journal for the European Communities*.

[29] Heleno S. A., Stojković D., Barros L. (2013). A comparative study of chemical composition, antioxidant and antimicrobial properties of Morchella esculenta (L.) Pers. from Portugal and Serbia. *Food Research International*.

[30] Ercoli A., de Ruggieri P. (1953). The constitution of cerebrosterol, a hydroxycholesterol isolated from horse brain. *Journal of the American Chemical Society*.

[31] Ward O. P., Young C. S. (1990). Reductive biotransformations of organic compounds by cells or enzymes of yeast. *Enzyme and Microbial Technology*.

[32] Singer Y., Shity H., Bar R. (1991). Microbial transformations in a cyclodextrin medium. Part 2. Reduction of androstenedione to testosterone by *Saccharomyces cerevisiae*. *Applied Microbiology and Biotechnology*.

[33] Długonski J., Wilmanska D. (1998). Deleterious effects of androstenedione on growth and cell morphology of Schizosaccharomyces pombe. *Antonie Van Leeuwenhoek*.

[34] Pajic T., Vitas M., Zigon D., Pavko A., Kelly S. L., Komel R. (1999). Biotransformation of steroids by the fission yeast Schizosaccharomyces pombe. *Yeast*.

[35] Kristan K., Rižner T. L. (2012). Steroid‐transforming enzymes in fungi. *The Journal of Steroid Biochemistry and Molecular Biology*.

[36] Park E. Y., Koike Y., Higashiyama K., Fujikawa S., Okabe M. (1999). Effect of nitrogen source on mycelial morphology and arachidonic acid production in cultures of *mortierella alpina*. *Journal of Bioscience and Bioengineering*.

[37] Jin M.-J., Huang H., Xiao A.-H. (2008). A novel two-step fermentation process for improved arachidonic acid production by *Mortierella alpina*. *Biotechnology Letters*.

[38] Zhang G.-P., Zhang F., Ru W.-M., Han J.-R. (2010). Solid-state fermentation of cornmeal with the ascomycete *Morchella esculenta* for degrading starch and upgrading nutritional value. *World Journal of Microbiology and Biotechnology*.

[39] Strobel R. J., Sullivan G. R. (1999). Experimental design for improvement of fermentations. *Manual of Industrial Microbiology and Biotechnology*.

[40] Reihani S. F. S., Khosravi-Darani K. (2019). Influencing factors on single cell protein production by submerged fermentation: a review. *Electronic Journal of Biotechnology*.

[41] Anupama, Ravindra P. (2000). Value-added food: single cell protein. *Biotechnology Advances*.

[42] Ravindra P., Alvarado P., Becerra M., Bilbao T., Moreau P. A. (2014). Morchella fluvialis sp. nov.(Ascomycota, Pezizales): a new but widespread morel in Spain. *Boletín de la Sociedad Micológica de Madrid*.

[43] García-Pascual P., Sanjuán N., Melis R., Mulet A. (2006). *Morchella esculenta* (morel) rehydration process modelling. *Journal of Food Engineering*.

[44] LeDuy A., Kosaric N., Zajic J. E. (1974). Morel mushroom mycelium growth in waste sulfite liquors as source of protein and flavouring. *Canadian Institute of Food Science and Technology Journal*.

[45] Aletor V. (1995). Compositional studies on edible tropical species of mushrooms. *Food Chemistry*.

[46] Bano Z., Rajarathanam S., Chang S. T., Quimio T. H. (1982). Pleurotus mushrooms as a nutritious food. *Tropical Mushrooms –Biological Nature and Cultivation Methods*.

[47] Roy A., Samajpati N. (1978). Agaricales of West Bengal II. *Indian Journal of Mushroom Research*.

[48] Chang S. T. (1980). Mushroom as human food. *Bio Science*.

[49] Li G. S. F., Chang S. T., Chang S. T., Quimio T. H. (1982). Nutritive value of Volvariella volvacea. *Tropical Mushrooms—Biological Nature and Cultivation Methods*.

[50] Crisan E. V., Sands A., Chang S. T., Hayes W. A. (1978). Nutritional value. *The Biology and Cultivation of Edible Mushrooms*.

[51] Hayes W. A., Haddad N. (1976). The food value of the cultivated mushrooms and its importance in industry. *Journal of Mushroom*.

[52] Gray J., Groeschler S., Le T., Gonzalez Z. (2002). *Membrane Structure (SWF)*.

[53] Yilmaz N., Solmaz M., Türkekul İ., Elmastaş M. (2006). Fatty acid composition in some wild edible mushrooms growing in the middle black region of Turkey. *Food Chemistry*.

[54] Hasan H. A. (1994). Production of hormones by fungi. *Acta Microbiologica Polonica*.

[55] Fernández-Cabezón L., Galán B., García J. L. (2017). Engineering *Mycobacterium smegmatis* for testosterone production. *Microbial Biotechnology*.

[56] Rowland I., Wiseman H., Sanders T., Adlercreutz H., Bowey E. (1999). Metabolism of oestrogens and phytoestrogens: role of the gut microflora. *Biochemical Society Transactions*.

[57] Clavel T., Lippman R., Gavini F., Doré J., Blaut M. (2007). Clostridium saccharogumia sp. nov. and Lactonifactor longoviformis gen. nov., sp. nov., two novel human faecal bacteria involved in the conversion of the dietary phytoestrogen secoisolariciresinol diglucoside. *Systematic and Applied Microbiology*.

[58] Landete J. M., Gaya P., Rodríguez E. (2017). Probiotic bacteria for healthier aging: immunomodulation and metabolism of phytoestrogens. *BioMed Research International*.

[59] Verma R. N., Singh G. B., Bilgrami K. S. (1987). Fleshy fungal flora of N. E. H. India- I. Manipur and Meghalaya. *Indian Mushroom Science*.

